# A Generalized SDP Multi-Objective Optimization Method for EM-Based Microwave Device Design

**DOI:** 10.3390/s19143065

**Published:** 2019-07-11

**Authors:** Ying Liu, Qingsha S. Cheng, Slawomir Koziel

**Affiliations:** 1 Department of Electrical and Electronic Engineering, Southern University of Science and Technology, Shenzhen 518055, China; 2 School of Electronics and Information Engineering, Harbin Institute of Technology, Harbin 150001, China; 3 School of Science and Engineering, Reykjavik University, Menntavegur 1, IS-101 Reykjavik, Iceland; 4 Faculty of Electronics, Telecommunications and Informatics, Gdansk University of Technology, 80-233 Gdansk, Poland

**Keywords:** multi-objective optimization, Pareto front, microwave devices design, EM-based simulation, generalized sequential domain patching, UWB antenna

## Abstract

In this article, a generalized sequential domain patching (GSDP) method for efficient multi-objective optimization based on electromagnetics (EM) simulation is proposed. The GSDP method allowing fast searching for Pareto fronts for two and three objectives is elaborated in detail in this paper. The GSDP method is compared with the NSGA-II method using multi-objective problems in the DTLZ series, and the results show the GSDP method saved computational cost by more than 85% compared to NSGA-II method. A diversity comparison indicator (DCI) is used to evaluate approximate Pareto fronts. The comparison results show the diversity performance of GSDP is better than that of NSGA-II in most cases. We demonstrate the proposed GSDP method using a practical multi-objective design example of EM-based UWB antenna for IoT applications.

## 1. Introduction

Nowadays, full-wave electromagnetic (EM) analysis has become a necessary tool for designing microwave devices. Full-wave EM analysis methods include method of moment (MoM) [1] and the finite-difference time domain method (FDTD) [2], et al. At present, microwave device models can be analyzed by commercial full-wave EM-based simulation software packages such as CST and HFSS. The full-wave EM analysis process is computationally expensive, especially for complex models. When the model is designed using numerical optimization methods such as the quasi-Newton method [3], genetic algorithm [4] or other population-based metaheuristic methods, the cost may become prohibitive.

In the design process of microwave devices, such as multi-port component design, many design objectives need to be considered at the same time, such as return loss [5], insertion loss [6] and so on. In many cases, especially IoT component design, the miniaturization of devices is also an objective that a designer has to consider [7]. Therefore, the design process can be seen as a multi-objective problem that takes many design objectives into consideration. These objectives require simultaneous optimization and are usually in conflict with each other, which means that improving one objective may degrade other objectives [8].

The commercial EM-based simulation software packages (CST [9] and HFSS [10]) allow the inclusion of many optimization objectives. Traditionally, these objectives can be converted into a single objective by weighted sum and then optimized by popular methods such as the genetic algorithm and pattern search method [11]. However, this method can only find one of many possible solutions, and the solution found by the arbitrary setting of the weight of each objective may not be satisfactory. Currently, the most robust multi-objective optimization algorithms with good convergence are population-based metaheuristic multi-objective optimization methods (such as NSGA-II [12]) and multi-objective particle swarm optimization methods [13]. In the case of EM-based component design [14,15,16] (each function evaluation normally requires a full-wave EM analysis), these population-based metaheuristic methods are not applicable because of the large amount of objective function evaluation required and thus the very high computation cost. Multi-objective optimization can also be carried out by constructing surrogate models of EM-based simulation of microwave devices [17,18,19,20] and combining with optimization algorithms such as NSGA-II. The computational cost is saved, but the accuracy of the solutions may suffer.

The sequential domain patching (SDP) method [21] overcomes the problem of a large number of function evaluations (EM simulations) by means of searching small areas (patch) sequentially between two optimal solutions. Since the SDP method uses EM simulation directly for multi-objective optimization, the error is relatively small. However, the SDP method is only applicable to two-objective optimization problems.

In this work, a generalized SDP (GSDP) method is proposed to efficiently solve EM-based multi-objective optimization. The method is applicable to solve optimization problems of two or three objectives, and it has the potential to be expanded to more objectives. Compared with the existing algorithms such as NSGA-II [12], the GSDP greatly reduces the computational cost and ensures a good diversity of optimization results.

## 2. GSDP Algorithm

### 2.1. Problem Formulation

Let *F_k_*(***x***), *k* = 1, …, *N_obj_*, be the *k*th design objective (***x*** stands for a vector of design parameters). The aim of the multi-objective optimization is to find an approximate Pareto set ***X*** so that for any ***x*** ∈ ***X***, there is no ***y*** for which ***y*** ≺ ***x***. Here, ≺ is the dominance relation defined as ***y*** ≺ ***x*** (***y*** dominates over ***x***) if *F_k_*(***y***) ≤ *F_k_*(***x***) for all *k* = 1, …, *N_obj_*, and *F_k_*(***y***) < *F_k_*(***x***) for at least one *k* [22]. A Pareto set in design space corresponds to a Pareto front [23] in the objective space. All the design solutions in ***X*** have the best possible trade-offs between the considered objectives.

### 2.2. Framework of GSDP Algorithm

The GSDP algorithm is designed to solve multi-objective problems of two or three objectives. The algorithm consists of four parts. The first step is to find all the extreme Pareto-optimal design solutions. Secondly, the Pareto set boundaries between every two extreme Pareto-optimal design solutions are found through an updated sequential domain patch (SDP [21]) method. If the problem is a two-objective optimization problem, the obtained boundary is defined as a “design profile”. The profile is then refined to obtain the final approximate Pareto set.

**Figure 1:**
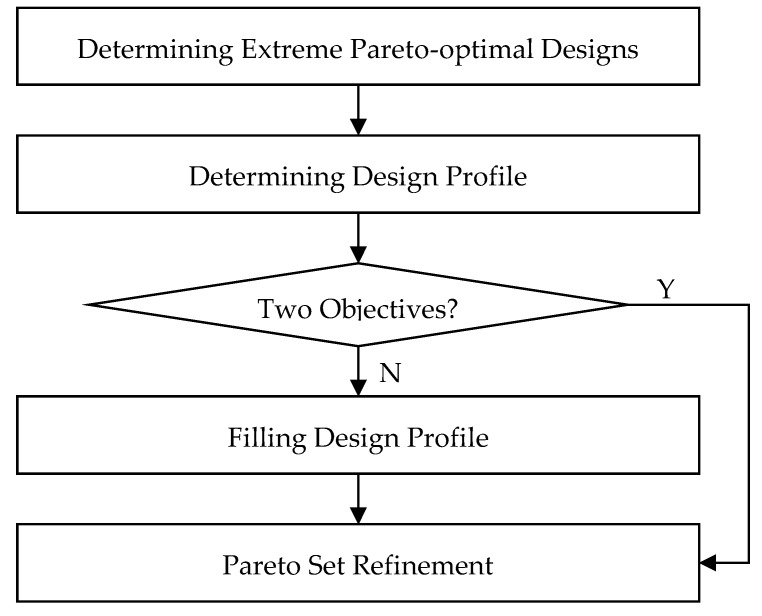
Algorithm flowchart of generalized sequential domain patching (GSDP).

If the problem is a three-objective optimization problem, the three Pareto set boundaries form a design profile. The profile is then filled with Pareto-optimal design solutions using the updated SDP method. These Pareto-optimal design solutions are refined to obtain the final approximate Pareto set. The flowchart of the GSDP is shown in Figure 1.

### 2.3. Determining Extreme Pareto-Optimal Designs

The GSDP algorithm begins by obtaining the extreme Pareto-optimal designs [24]. Here, the extreme pareto-optimal designs are a set of optimal solutions for all single objectives. The optimal solution $x_{k}^{*}$ for *k*th objective, *k* = 1, …, *N_obj_*, is defined as follows:

(1) $$x_{k}^{*}=\underset{x}{\arg\min}F_{k}(x)$$

### 2.4. Determining Design Profile

After all the extreme Pareto-optimal designs are obtained, we use an updated SDP method to obtain the Pareto set boundaries between every two extreme Pareto-optimal designs. There is only one Pareto set boundary which can be obtained when it is a two-objective problem, and this boundary forms a so-called design profile. When it is a three-objective problem, all three Pareto set boundaries are linked together to form a design profile. This process of finding a Pareto set boundary is equivalent to solving a two-objective optimization problem.

Suppose a two-objective optimization problem is defined as follows:

(2) $$F=\left\{ \begin{array}{l} \min F_{1}(x)\\ \min F_{2}(x) \end{array}\right.,$$

where x is the *n*-dimensional design parameter vector. $x_{1}^{*}$ and $x_{2}^{*}$ are the single objective optimal solutions of *F*_1_ and *F*_2_, respectively, 

(3) $$x_{1}^{*}=\underset{x}{\arg\min}F_{1}(x),$$

(4) $$x_{2}^{*}=\underset{x}{\arg\min}F_{2}(x).$$

The method to find the Pareto set boundary of the two objectives is described in detail as follows:

1. Determine the design space; the lower bound vector ***lb*** and upper bound vector ***ub*** can be defined as follows:
  (5) $$lb_{j}=\min(x_{1j}^{*},x_{2j}^{*}),$$
  (6) $$ub_{j}=\max(x_{1j}^{*},x_{2j}^{*}),$$
  where $lb_{j}$ and $ub_{j}$ are the *j*th variables of vectors ***lb*** and ***ub*** respectively; $x_{1j}^{*}$ and $x_{2j}^{*}$ are the *j*th variables of vectors $x_{1}^{*}$ and $x_{2}^{*}$ respectively.
2. Set the perturbation size vector ***d*** = [*d*_1_, …, *d_n_*]*^T^*.
3. Let the current points $x_{c}^{1}=x_{1}^{*}$ and $x_{c}^{2}=x_{2}^{*}$.
4. Evaluate *n* perturbations $x_{k}^{p}=x_{c}^{1}+{\left[0,\ldots ,0,d_{k},0,\ldots ,0\right]}^{T}$ (*k* = 1, *…*, *n*) around $x_{c}^{1}$ (towards $x_{c}^{2}$ only) and select the one that leads to the largest improvement with regard to the second objective *F*_2_ as the current point $x_{c}^{1}$.
5. Evaluate *n* perturbations $x_{k}^{p}=x_{c}^{2}+{\left[0,\ldots ,0,d_{k},0,\ldots ,0\right]}^{T}$ (*k* = 1, *…*, *n*) around $x_{c}^{2}$ (towards $x_{c}^{1}$ only) and select the one that leads to the largest improvement with regard to the first objective *F*_1_ as the current point $x_{c}^{2}$.
6. If $x_{c}^{1}$ or $x_{c}^{2}$ exceeds the design space, the process is stopped. If both $x_{c}^{1}$ and $x_{c}^{2}$ are in the design space, return to step 4.

In step 2, the perturbation size vector ***d*** can be obtained by the following formula:

(7) $$d_{j}=\frac{\left|x_{1j}^{*}-x_{2j}^{*}\right|}{K},$$

where $d_{j}\;(j=1,\ldots ,n)$ means the size of the perturbations in the *j*th direction. The value of *K* is a user-defined constant (typically between 5 and 20).

**Figure 2:**
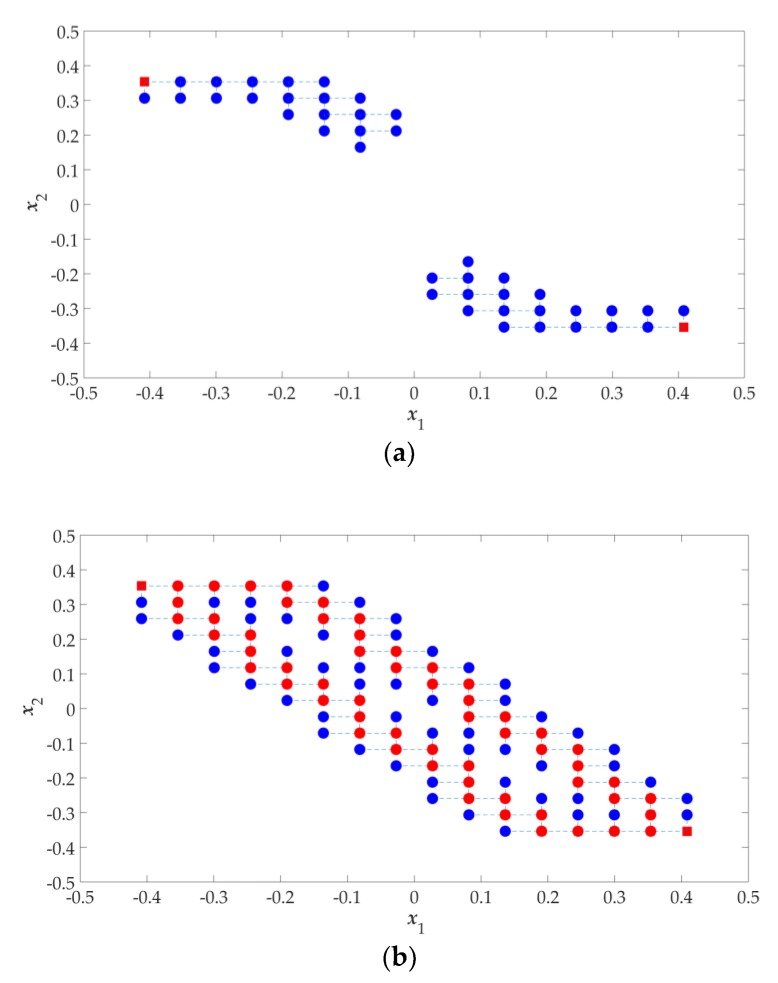
Illustration of the process of finding a Pareto set boundary by using an updated SDP method: (**a**) Starting points (red square) and perturbation points (blue dot, where the best perturbations become the centers of the next patches) of the first 10 iterations; (**b**) Pareto set boundary (red points) found.

**Figure 3:**
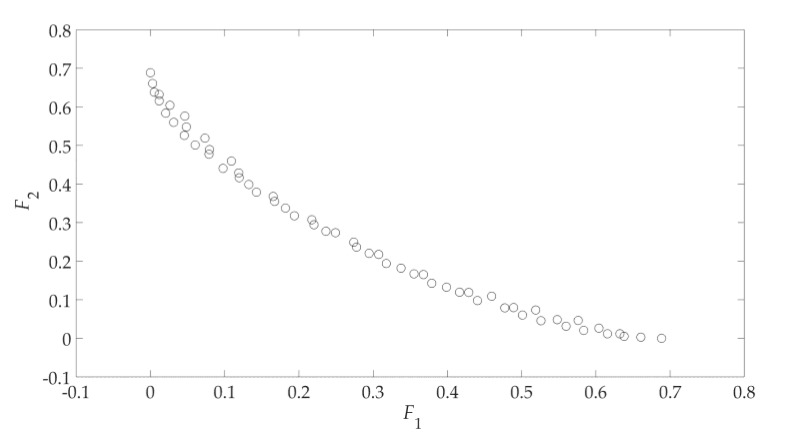
The Pareto front boundary in the objective space.

As an illustration, the above method is applied to solve a two-objective optimization problem:

(8) $$F=\left\{ \begin{array}{l} \min f_{1}(x)=1-e^{-{\left(x_{1}-\frac{1}{\sqrt{6}}\right)}^{2}-{\left(x_{2}+\frac{1}{\sqrt{8}}\right)}^{2}}\\ \min f_{2}(x)=1-e^{-{\left(x_{1}+\frac{1}{\sqrt{6}}\right)}^{2}-{\left(x_{2}-\frac{1}{\sqrt{8}}\right)}^{2}} \end{array}\right.,$$

where the design parameter dimension *n* = 2 and constant *K* = 15; the process to find the Pareto set boundary is shown in Figure 2. Corresponding to the Pareto set boundary for the two-objective problem in Figure 2, the Pareto front boundary in the objective space is shown in Figure 3.

**Figure 4:**
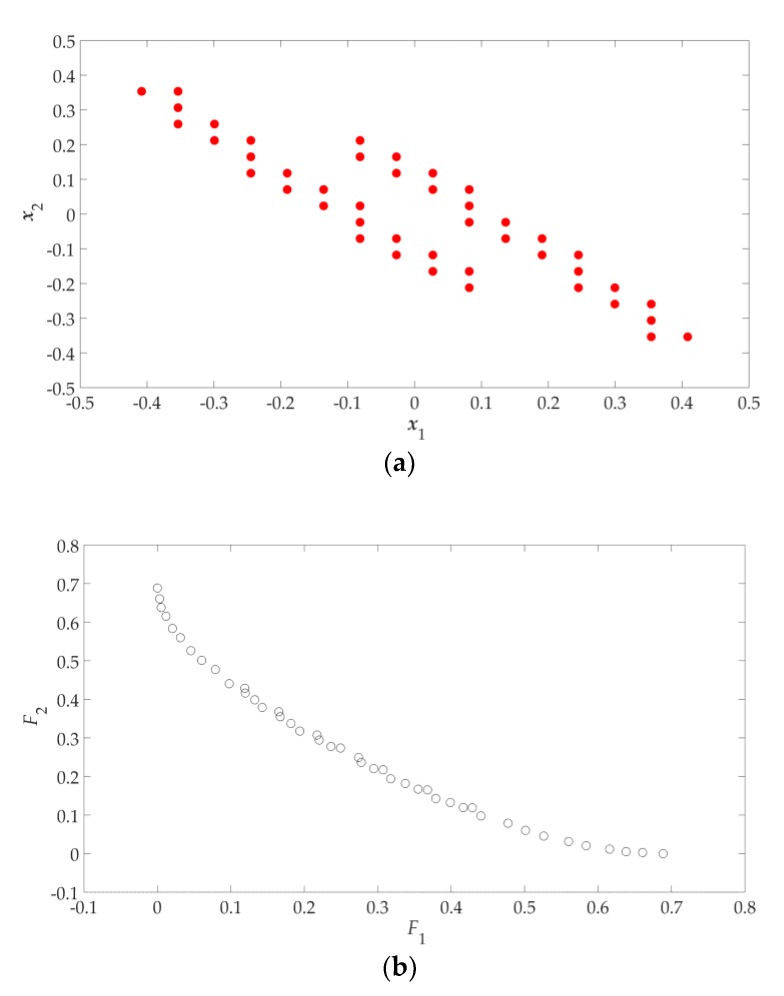
Results of two design objectives: (**a**) approximate Pareto set (design space); (**b**) approximate Pareto front (objective space).

Since (8) is a two-objective optimization problem, the design profile is formed by the Pareto set boundary (shown as red dots in Figure 2b). Its corresponding Pareto front boundary (shown in Figure 3) is called the “objective profile”. According the GSDP algorithm flowchart (shown in Figure 1), all the solutions in the design profile need to be refined to obtain the final approximate Pareto set. Its corresponding approximate Pareto front is shown in Figure 4. The refinement process is described later in Section 2.6.

**Figure 5:**
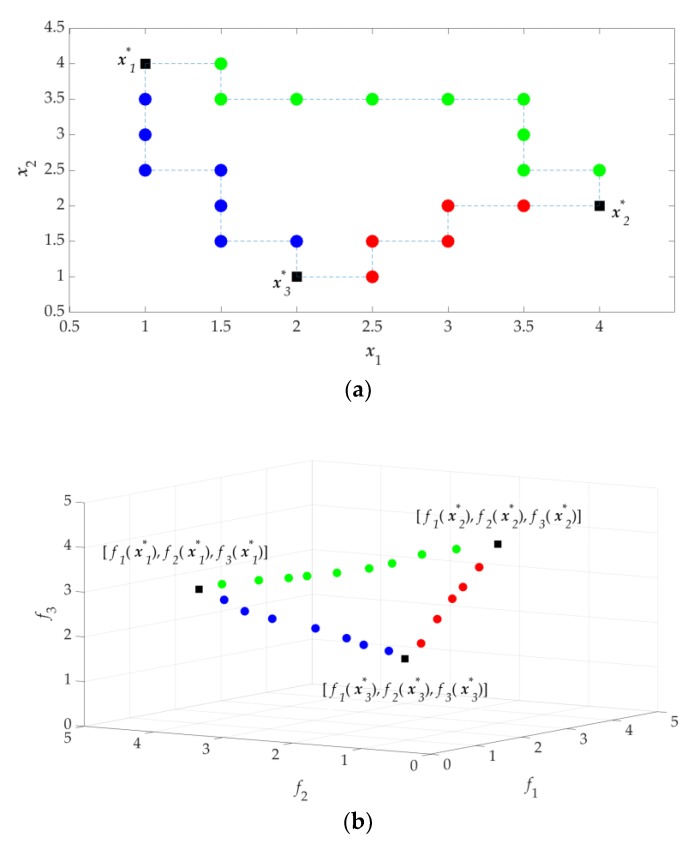
Conceptual illustration of three design objectives: (**a**) design profile (design space); (**b**) objective profile (objective space).

If the problem is a three-objective optimization problem, the extreme Pareto-optimal designs ***X***_12_, ***X***_13_, and ***X***_23_ are obtained first, where ***X****_ij_* is the Pareto set boundary of the *i*th and *j*th objectives found by updated SDP, and ***F****_ij_* is the Pareto front boundary corresponding to ***X****_ij_*. ***F***_12_, ***F***_13_ and ***F***_23_ form the objective profile for three objectives. The conceptual results for the obtained design profile and objective profile for three objectives are illustrated in Figure 5.

### 2.5. Filling Design Profile

In the case of three design objectives ($f_{1}$, $f_{2}$ and $f_{3}$), the corresponding extreme optimal designs are $x_{1}^{*}$, $x_{2}^{*}$ and $x_{3}^{*}$, respectively. The design profile found following Section 2.4 is filled by the process described below.

**Figure 6:**
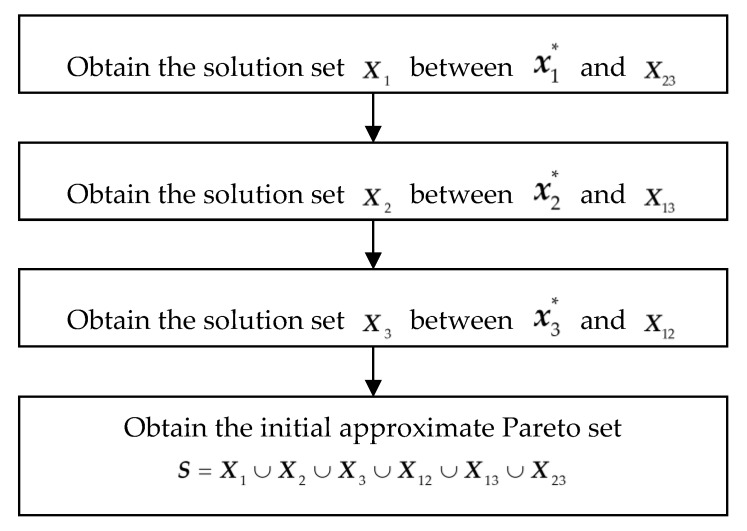
Process of filling the design profile.

The process finds the appropriate design set $X_{i}$ between $x_{i}^{*}$ and its opposite Pareto set boundary ***X****_jk_* (*j*, *k =* 1, 2, 3 and *j*, *k* ≠ *i*) for all *i* = 1, 2, 3. The design sets $X_{i}$ (*i* = 1, 2, 3) and the design profile are combined to form the initial approximate Pareto set ***S***. In other words, the design profile is filled by the three similar steps shown in the flowchart in Figure 6. 

Here, we only describe the process of obtaining the solution set $X_{1}$ between $x_{1}^{*}$ and $X_{23}$ for ease of reading. The processes to find $X_{2}$ and $X_{3}$ are similar.

1. Let $X_{23}^{i}$ be the *i*th solution in $X_{23}$, i=1.
2. If *i* > *N*, the process stops, where *N* is the number of solutions in $X_{23}$.
3. Determine the design subspace, the lower bound vector ***lb*** and upper bound vector ***ub*** can be defined as follows:
  (9) $$lb_{j}=\min(x_{1j}^{*},X_{23_{j}}^{i}),$$
  (10) $$ub_{j}=\max(x_{1j}^{*},X_{23_{j}}^{i}),$$
  where $lb_{j}$ and $ub_{j}$ are the *j*th variables of vectors ***lb*** and ***ub***, respectively, and $x_{1j}^{*}$ and $X_{23_{j}}^{i}$ are the *j*th variables of vectors $x_{1}^{*}$ and $X_{23}^{i}$, respectively, where $X_{23}^{i}$ is the *i*th solution in $X_{23}$.
4. Set the perturbation size vector ***d*** = [*d*_1_, …, *d_n_*]*^T^*, $d_{j}=\frac{\left|x_{1j}^{*}-X_{23_{j}}^{i}\right|}{K}$; *K* is up to the user.
5. Let the current points $x_{c}^{1}=x_{1}^{*}$ and $x_{c}^{2}=X_{23}^{i}$.
6. Evaluate *n* perturbations $x_{k}^{p}=x_{c}^{1}+{\left[0,\ldots ,0,d_{k},0,\ldots ,0\right]}^{T}$ (*k* = 1, *…*, *n*) around $x_{c}^{1}$ (towards $x_{c}^{2}$ only) and select the one that leads to the largest improvement with regard to $F_{2}=a*f_{2}+b*f_{3}$ as the current point $x_{c}^{1}$.
7. Evaluate *n* perturbations $x_{k}^{p}=x_{c}^{2}+{\left[0,\ldots ,0,d_{k},0,\ldots ,0\right]}^{T}$ (*k* = 1, *…*, *n*) around $x_{c}^{2}$ (towards $x_{c}^{1}$ only) and select the one that leads to the largest improvement with regard to $F_{1}=f_{1}$ as the current point $x_{c}^{2}$.
8. If $x_{c}^{1}$ and $x_{c}^{2}$ exceeds the design subspace, i=i+1, return to step 2. If both $x_{c}^{1}$ and $x_{c}^{2}$ are still in the design space, return to step 6.

**Figure 7:**
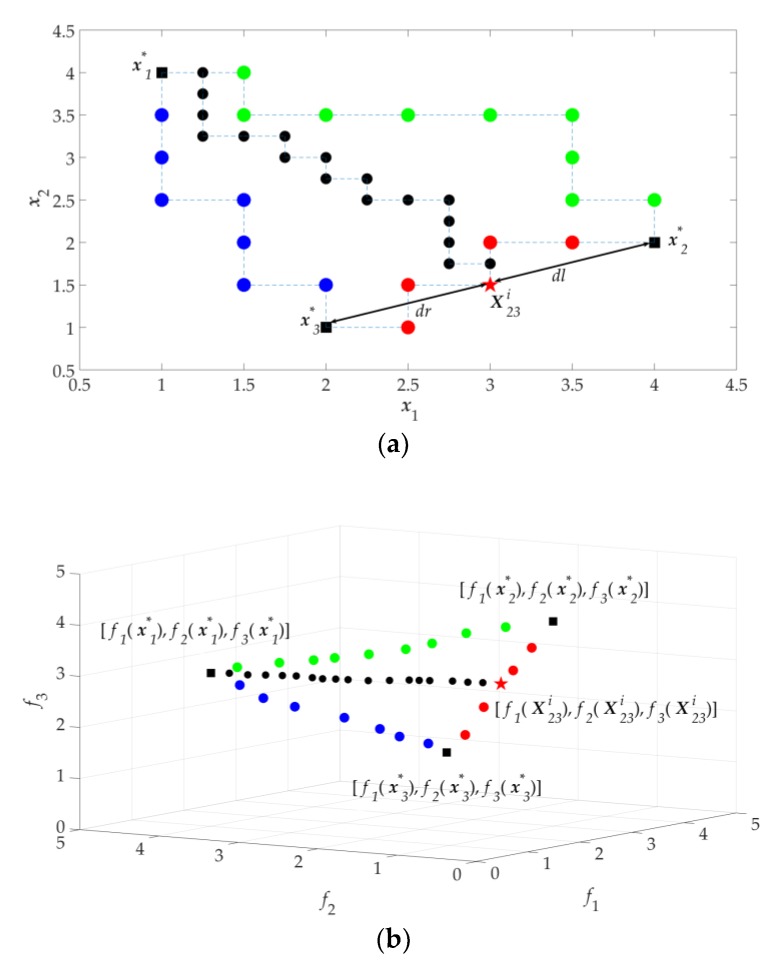
Filling the path between $x_{1}^{*}$ and $X_{23}^{i}$. (**a**) Design space. The red five-pointed star is $X_{23}^{i}$, and the black dots are the path between $x_{1}^{*}$ and $X_{23}^{i}$. (**b**) Objective space. The red star is the corresponding representation of $X_{23}^{i}$ in the objective space, and the black dots are the corresponding representation of the path in the objective space.

The weighting factors *a* and *b* in step 6 are defined as follows:

(11) $$a=\frac{dl}{dl+dr},$$

(12) $$b=\frac{dr}{dl+dr},$$

where *dl* is the distance between $X_{23}^{i}$ and $x_{2}^{*}$ in the design space, and *dr* is the distance between $X_{23}^{i}$ and $x_{3}^{*}$. When $x_{1}^{*}$ and $X_{23}^{i}$ are taken as starting points to locate set $X_{1}$ by the process mentioned above, the relative location of $X_{23}^{i}$ in $X_{23}^{}$ affects the selection of the perturbed design. As shown in Figure 7a, if *dl* < *dr*, the selection is biased towards $x_{3}^{*}$ and if *dl* > *dr*, the selection is biased towards $x_{2}^{*}$. Therefore, the weighting factors *a* and *b* are necessary to balance the bias. The process to connect $x_{1}^{*}$ and $X_{23}^{i}$ is illustrated as Figure 7.

When the filling design profile is complete, all the obtained paths and design profiles obtained in Section 2.4 together constitute the initial approximate Pareto set.

### 2.6. Pareto Set Refinement

In the above method, the initial approximate Pareto set and the corresponding Pareto front are obtained, so there are still many dominated solutions in the result. These dominated solutions are deleted to ensure the results are all non-dominated solutions (the final approximation Pareto set).

### 2.7. Evaluation

The Diversity Comparison Indicator (DCI) [25] is used to assess the diversity of the obtained Pareto front with respect to the other Pareto fronts. In this method, all the concerned Pareto fronts are compared together and the dominated solutions are removed. The remaining solutions are put into a grid environment. DCI considers the total contribution of each Pareto front to all the nonempty grid cells. The DCI method can only be applied to the assessment of relative diversity of a Pareto front against others rather than providing an absolute measure of distribution for a single Pareto front. The DCI value is a number in the range [0, 1], and the higher the DCI value, the better the distribution of this Pareto front.

## 3. Illustration and Verification Examples

In this section, we use 4 DTLZ three-objective functions to verify the GSDP algorithm.

### 3.1. Design Case 1: DTLZ1

The DTLZ1 [26] function is as follows:

(13) $$DTLZ1=\left\{ \begin{array}{l} \min f_{1}(x)=0.5x_{1}x_{2}(1+g(x))\\ \min f_{2}(x)=0.5x_{1}(1-x_{2})(1+g(x))\\ \min f_{3}(x)=0.5(1-x_{1})(1+g(x))\\ g(x)=100\left(n-2+\sum_{i=3}^{n}\left({\left(x_{i}-0.5\right)}^{2}-\cos\left(20\pi \left(x_{i}-0.5\right)\right)\right)\right) \end{array}\right.,$$

where $x_{i}$ means the *i*th variable of *n*-dimensional vector parameter ***x***.

Here, parameter ***x*** has a dimension of *n* = 6. The built-in function in MATLAB *fmincon* is used with the initial optimization points [0, 0, 0, 1, 1, 1] to obtain the three single-objective optimal solutions, $x_{1}^{*}$ = [0.0008, 0.0008, 0.5000, 0.5000, 0.5000, 0.5000], $x_{2}^{*}$ = [0.1784, 0.8241, 0.5999, 0.4001, 0.4001, 0.4001], $x_{3}^{*}$ = [1.0000, 0.5404, 0.7998, 0.4002, 0.4002, 0.4000]. Then, let the three solutions be the starting points of GSDP. The approximate Pareto front of 233 solutions is obtained by GSDP, and the process costs 5303 $f_{1}$ function evaluations, 5394 $f_{2}$ function evaluations and 5218 $f_{3}$ function evaluations.

**Figure 8:**
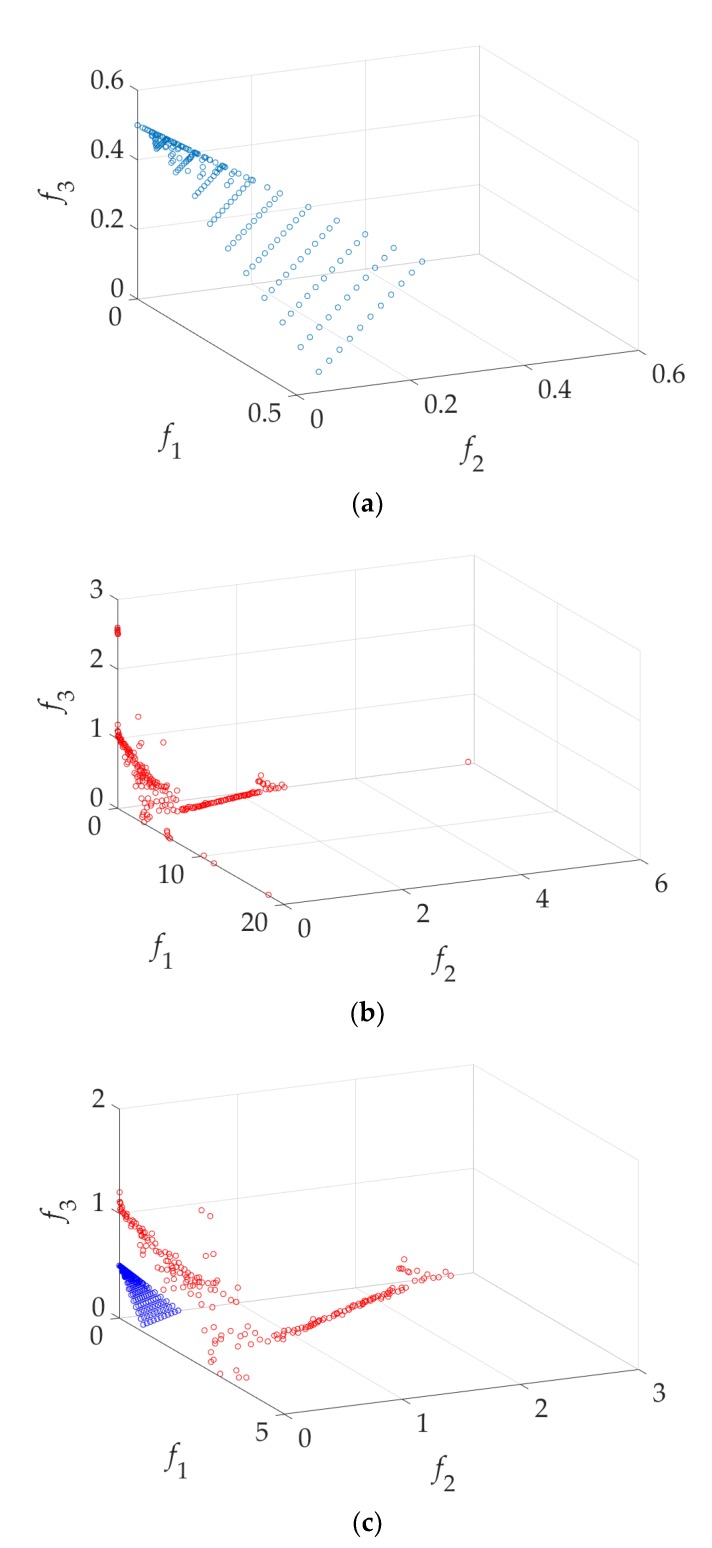
Approximate Pareto fronts of DTLZ1 (*n* = 6): (**a**) obtained by GSDP; (**b**) obtained by NSGA-II; (**c**) comparison between GSDP and NSGA-II.

As a comparison, (13) is also solved using the built-in function in MATLAB *gamultiobj* which is a controlled, elitist genetic algorithm (a variant of NSGA-II). To find the approximate Pareto front of 233 solutions, each of the objective functions ($f_{1}$, $f_{2}$, and $f_{3}$) is evaluated 105,735 times. The computational cost of GSDP is 95% less than that of NSGA-II. The approximate Pareto fronts P1 and P2 obtained by the GSDP and NSGA-II, respectively, are shown in Figure 8.

By combining P1 and P2 and deleting the dominated solutions, all the 233 solutions in the P1 are retained, while only 37 solutions in the P2 are retained. That means 196 solutions in P2 are dominated by the solutions in P1.

The distribution performances of the obtained two Pareto fronts are evaluated by the Diversity Comparison Indicator (DCI) [25]. In this case, the DCI value of P1 is 0.7667, while the DCI value of P2 is 0.2333.

As demonstrated in this example, GSDP is much more efficient than NSGA-II. Moreover, the distribution of the approximate Pareto front of GSDP is relatively better than that of NSGA-II.

### 3.2. Design Case 2: DTLZ2 

The DTLZ2 [26] function is as follows:

(14) $$DTLZ2=\left\{ \begin{array}{l} \min f_{1}(x)=\cos(\frac{\pi }{2}x_{1})\cos(\frac{\pi }{2}x_{2})(1+g(x))\\ \min f_{2}(x)=\cos(\frac{\pi }{2}x_{1})\sin(\frac{\pi }{2}x_{2})(1+g(x))\\ \min f_{3}(x)=\sin(\frac{\pi }{2}x_{1})(1+g(x))\\ g(x)=\sum_{i=3}^{n}{\left(x_{i}-0.5\right)}^{2} \end{array}\right.,$$

where $x_{i}$ means the *i*th variable of the n-dimensional vector parameter ***x***.

At *n* = 9, the built-in function in MATLAB *fmincon* is used with the initial optimization points [0, 0, 0, 1, 1, 0, 0, 1, 0] to obtain the three single-objective optimal solutions $x_{1}^{*}$ = [0.9994, 0.9994, 0.5038, 0.4962, 0.4962, 0.5038, 0.5038, 0.4962, 0.5038], $x_{2}^{*}$ = [0.9524, 0.0077, 0.5004, 0.4996, 0.4996, 0.5004, 0.5004, 0.4996, 0.5004], $x_{3}^{*}$ = [0.0000, 0.4907, 0.5069, 0.4931, 0.4931, 0.5069, 0.5069, 0.4931, 0.5069]. Then, let the three solutions be the starting points of GSDP. The approximate Pareto front of 833 solutions is obtained by GSDP, and the process costs 6328 $f_{1}$ function evaluations, 9208 $f_{2}$ function evaluations and 6270 $f_{3}$ function evaluations.

**Figure 9:**
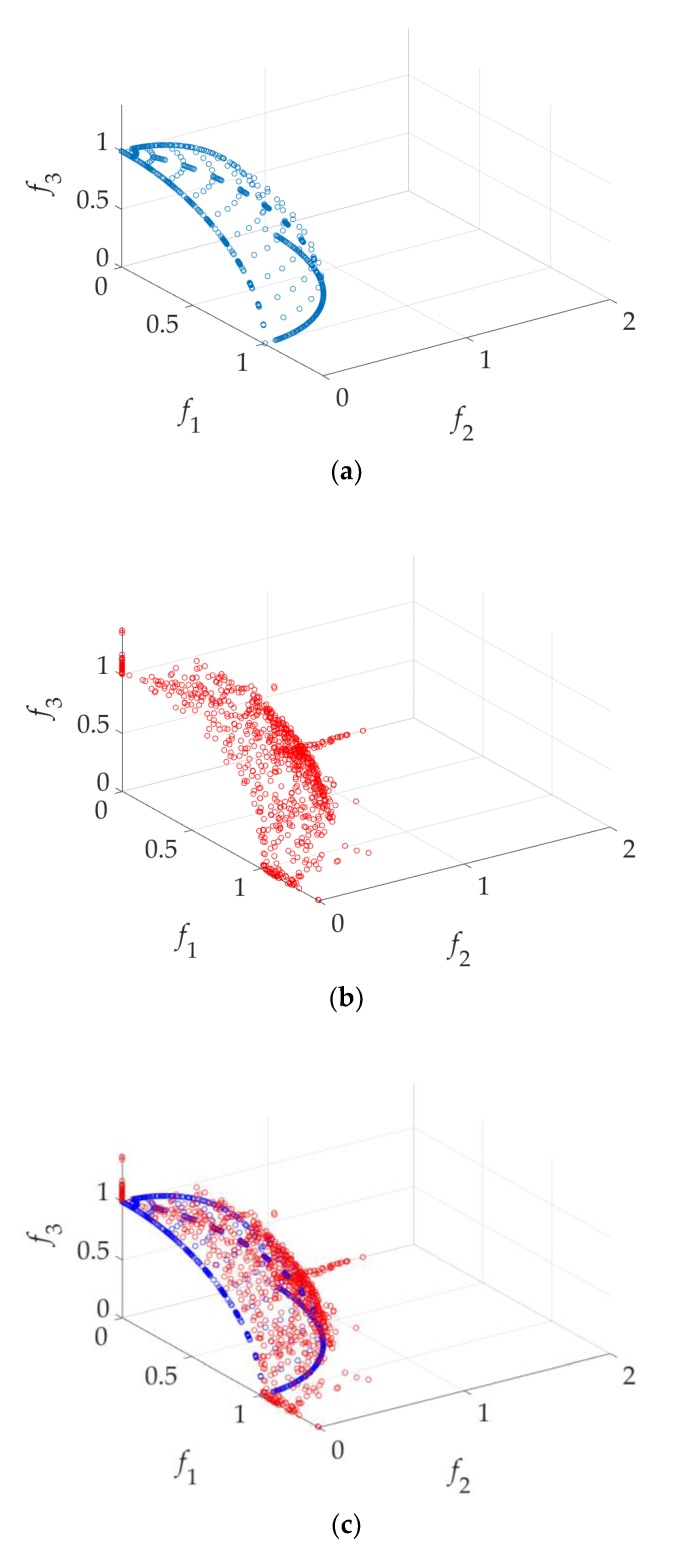
Approximate Pareto fronts of DTLZ2 (*n* = 9): (**a**) obtained by GSDP; (**b**) obtained by NSGA-II; (**c**) comparison between GSDP and NSGA-II.

As a comparison, NSGA-II is used to obtain 833 solutions of the approximate Pareto front. Each objective function is evaluated 268,940 times. GSDP saves about 97% of computational cost compared to NSGA-II. The approximate Pareto fronts P1 and P2 obtained by the GSDP and NSGA-II, respectively, are shown in Figure 9. 

After combining P1 and P2 and deleting the dominated solutions, all 833 solutions in the P1 are retained, while only 454 solutions in the P2 are retained. The DCI values of P1 and P2 are 0.4840 and 0.5426, respectively. Although the DCI of P1 is slightly less than that of P2, the GSDP has absolute advantages in terms of computational cost.

### 3.3. Design Case Validation Summary

In addition to the above test results, the GSDP method is tested using more three-objective functions with variables of various dimensions. We summarize the results in Table 1. P1 and P2 represent the approximate Pareto fronts obtained by the GSDP and NSGA-II, respectively. P1 and P2 have the same number of solutions. It can be seen that for a variety of problems and variable dimensions, the GSDP method performs better than or similar to NSGA-II in terms of distribution, but GSDP saves more than 85% in computational cost for all the testing cases. 

## 4. UWB Antenna Multi-Objective Design Example

**Figure 10:**
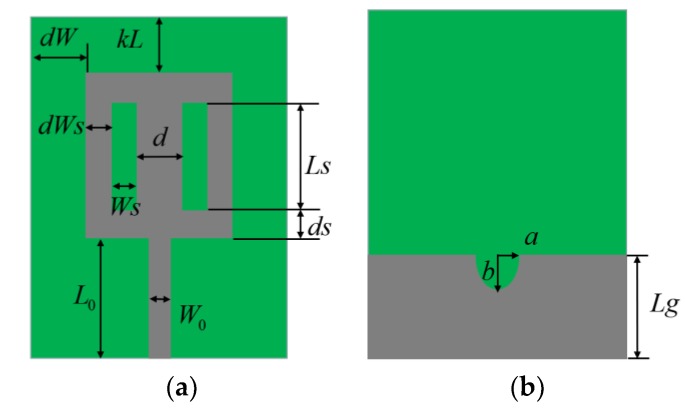
UWB antenna structure: (**a**) the front view; (**b**) back view. The gray part is the metal patch and the green part is the dielectric substrate.

The proposed GSDP method is applied to the multi-objective optimization of a microwave device: a UWB antenna [27]. The antenna is implemented on an FR4 substrate (*ε* = 4.3, *h* = 1.55 mm, *tan*δ = 0.02). Here, $f_{1}$ (the first objective) is used to minimize $\left|S_{11}\right|(\mathrm{dB})$ between 4 GHz and 10 GHz, while $f_{2}$ (the second objective) is used to minimize the difference of the realized gain between 4 GHz and 10 GHz and $f_{3}$ (the third objective) is used to minimize the UWB antenna size. The antenna structure and design parameter vector $x=[a,b,d,kL,ds,dW,dWs,L_{0},Lg,Ls,Ws]$ are shown in Figure 10.

**Figure 11:**
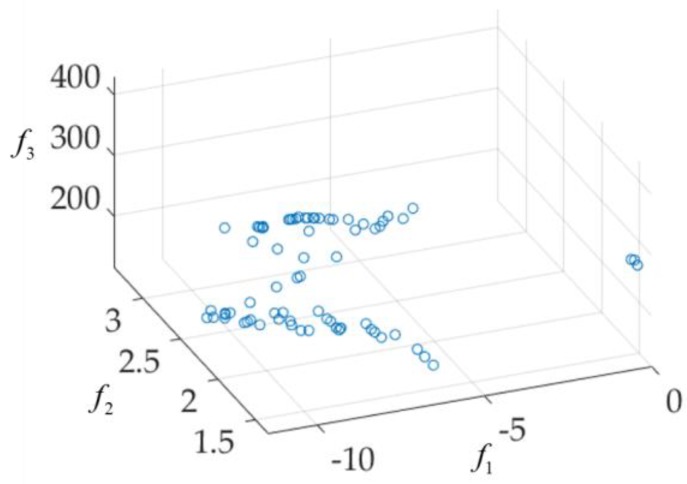
The approximate Pareto front found by GSDP.

Here, $W_{0}=0.731$ is fixed. The first step of the GSDP method is to find the three extreme optimal design solutions using single-objective optimization (here, a genetic algorithm is used): $x_{1}^{*}$ = [1.1149, 0.5310, 4.3989, 6.2208, 1.4515, 2.3120, 1.3032, 12.5860, 8.6279, 8.0845, 0.5176], $x_{2}^{*}$ = [0.5991, 0.4793, 4.9893, 6.4903, 1.7809, 2.0737, 2.5576, 9.5941, 6.2921, 8.8158, 0.4254], $x_{3}^{*}$ = [1.1813, 0.3480, 2.3139, 3.6562, 0.6466, 1.1315, 1.1168, 5.3947, 3.6096, 5.3438, 0.2531]. Then, the approximate Pareto front is found by GSDP as shown in Figure 11. 

**Figure 12:**
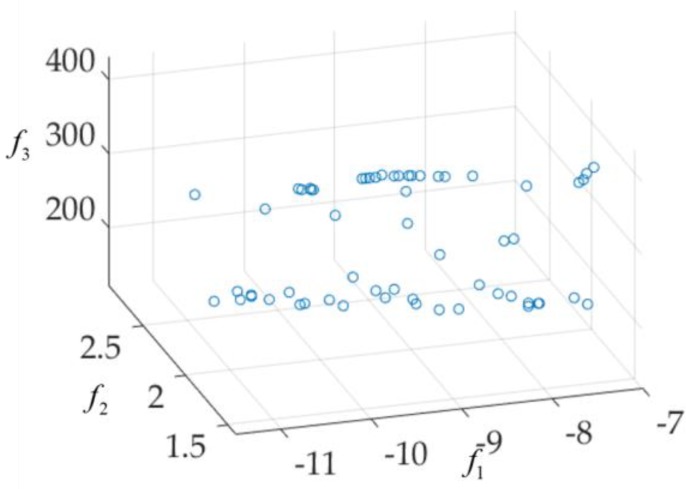
The applicable designs for the UWB antenna.

**Figure 13:**
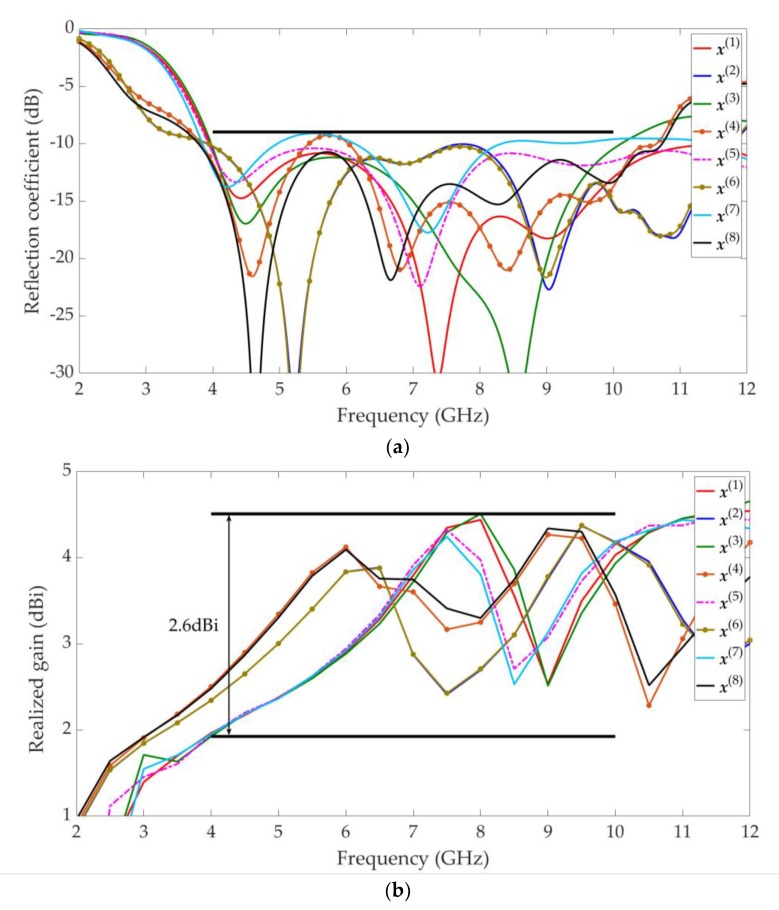
The selected design results ($x^{\left(1\right)}$, $x^{\left(2\right)}$, …, $x^{\left(8\right)}$ ref. Table 2) on the approximate Pareto set obtained by GSDP: (**a**) All $S_{11}(\mathrm{dB})$ responses are below −9 dB between 4 GHz and 10 GHz; (**b**) All the realized gain (dBi) variationd are below 2.6 between 4 GHz and 10 GHz.

Some of the solutions in the Pareto front shown in Figure 11 may not applicable to the design of UWB antennae. For example, the $f_{1}$ ($\left|S_{11}\right|$) value is greater than −7 dB. These solutions are discarded. Then, the final UWB antenna multi-objective design results are shown in Figure 12. Eight solutions ($x^{\left(1\right)}$, $x^{\left(2\right)}$, …, $x^{\left(8\right)}$) are selected according to customized specifications (e.g., all are below −9 dB between 4 GHz and 10 GHz, all the realized gain (dBi) variation are below 2.6 between 4 GHz and 10 GHz, and sizes are below 400 mm^2^). Their objective values and parameter values are shown in Table 2. The $S_{11}(\mathrm{dB})$ responses (reflection coefficient) and the realized gain (dBi) are shown in Figure 13.

A total of 3466 CST simulations are used to optimize the UWB antenna, which includes 1600 simulations to obtain the extreme optimal solutions (single-objective optimization process). This takes about 48.14 h on an Intel(R) Core i5-6500 processor with 8 GB RAM. If the NSGA-II method were used for the three-objective optimization of this UWB antenna (11 design variables), the computational cost is estimated at more than 100,000 CST EM simulations (see DTLZ2(11) in Table 1), which is prohibitive.

## 5. Conclusions

An efficient multi-objective optimization method (GSDP) for EM-based microwave device design is presented. The GSDP method generalizes the SDP method to solve multi-objective problems of both two and three objectives. The GSDP method includes four parts: (i) determining the extreme Pareto-optimal designs; (ii) determining the design profile; (iii) filling the design profile; and (iv) refining the Pareto set. Each part is described in detail. The GSDP method is compared to NSGA-II in terms of computational efficiency and the performances of Pareto fronts. The GSDP method saves more than 85% in computational cost compared to NSGA-II with similar or better performance in terms of Pareto front distribution (DCI). A UWB antenna multi-objective EM-based design example demonstrates the efficiency of GSDP in finding the approximate Pareto set (front). Selected designs from the approximate Pareto set satisfy all three customized specifications.

## Figures and Tables

**Table 1:** Comparison results of GSDP and NSGA-II methods. DCI: Diversity Comparison Indicator.

Functions (Dimension of Design Parameter)	Number of Solutions in P1 or P2	Function Evaluation Times Using GSDP (P1)	Function Evaluation Times Using NSGA-II (P2)	Time Saving	DCI (P1)	DCI (P2)
DTLZ1 (6)	233	15,915	317,205	95%	0.7667	0.2333
DTLZ2 (9)	833	21,806	806,820	97%	0.4840	0.5426
DTLZ1 (5)	470	13,299	232,650	94%	0.9424	0.0576
DTLZ2 (11)	348	10,043	124,236	92%	0.4737	0.5533
DTLZ3 (12)	1921	58,338	1,192,941	95%	0.9602	0.0398
DTLZ4 (7)	530	24,358	179,670	86%	0.4148	0.6049

**Table 2:** Objective values and parameter values of selected designs found by GSDP.

	$x^{\left(1\right)}$	$x^{\left(2\right)}$	$x^{\left(3\right)}$	$x^{\left(4\right)}$	$x^{\left(5\right)}$	$x^{\left(6\right)}$	$x^{\left(7\right)}$	$x^{\left(8\right)}$
$f_{1}$	−10.7307	−10.0570	−10.3724	−9.2783	−10.4177	−10.2159	−9.1132	−10.7473
$f_{2}$	2.4739	2.0232	2.5810	1.7619	2.369881	2.0293	2.2915	1.8562
$f_{3}$	145.9946	347.8369	140.0131	369.7517	164.1404	350.5260	162.1286	353.8818
*a*	1.1813	0.5991	1.1813	1.1149	1.1813	0.5991	1.1813	1.1149
*b*	0.3480	0.4793	0.3480	0.5310	0.3480	0.4793	0.3480	0.5310
*d*	3.0089	4.9893	2.3139	4.3989	2.3139	3.2057	2.3139	4.3989
*kL*	3.6562	6.4903	4.5111	6.2208	3.6562	6.4903	3.6562	6.2208
*ds*	0.9149	1.7809	0.6466	1.4515	0.6466	1.7809	0.6466	1.4515
*dW*	1.1315	2.0737	1.1315	2.3120	1.9185	2.0737	1.8398	1.9185
*dWs*	1.1168	1.1168	1.1168	1.3032	1.1789	2.0558	1.1168	1.3032
*L*_0_	7.3929	9.5941	7.0745	11.9876	7.9143	9.5941	8.2712	12.5860
*Lg*	3.6096	6.2921	3.6096	8.6279	3.6096	6.2921	3.6096	8.6279
*Ls*	5.3438	8.8158	6.2574	8.0845	5.3438	8.8158	5.3438	8.0845
*Ws*	0.2531	0.4254	0.2531	0.5176	0.2531	0.4254	0.2531	0.5176
